# Occurrence des traumatismes alvéolo-dentaires aux cliniques universitaires de Kinshasa: deuxième partie, étude préliminaire de 93 cas

**DOI:** 10.11604/pamj.2018.29.50.13763

**Published:** 2018-01-18

**Authors:** Adelin Nzudjom Foche, Fidele Nyimi Bushabu, Charles Mfutu Mana, Ramazani Haruna, Steve Sekele Masin, Monique Nsudila, Paul Bobe Alifi, Pierre Muyembi Muinamiyi, Jean Paul Sekele Isourady Burley

**Affiliations:** 1 Unité de Chirurgie Orale, Clinique Universitaires de Kinshasa, Kinshasa, République Démocratique du Congo; 2 Chirurgie Orale et Maxillo-Faciale, Cliniques Universitaires de Kinshasa, Kinshasa RD, Congo; 3 Hôpital Stomatologie de l’Université de Wuhan, Wuhan, Chine; 4 Département de Mathématique et Informatique, Faculté des Sciences de l’Université de Kinshasa, Kinshasa RD, Congo; 5 Service de Prothèse Dentaire et Maxillo-Faciale et Orthopeudi-Dentofaciale, Cliniques Universitaires de Kinshasa RD, Congo

**Keywords:** Profil clinique, fractures alvéolo-dentaires, Kinshasha

## Abstract

**Introduction:**

L'objectif de cette étude était d'analyser les caractéristiques épidémiologiques cliniques des fractures alvéolo-dentaires aux cliniques universitaires de Kinshasa/RDC.

**Méthodes:**

Étude transversale de 7 ans, réalisée dans le département d'odontostomatologie, service de stomatologie et de chirurgie maxillo-faciale/cliniques universitaires de Kinshasa de Janvier 2007 à Décembre 2014.

**Résultats:**

Sur les 93 dossiers colligés, le sexe masculin était prédominant (66,7%) et l'âge le plus prévalent se situait entre 20 ans et 29 ans. Les causes les plus fréquentes ont été les agressions/rixes (52,7%) et la luxation dentaire a été le type des lésions la plus fréquente avec 52,7%. La RX retro-alvéolaire été réalisée dans 75,7%, et le blocage mono-maxillaire fut le type de traitement le plus réalisé (60,2%).

**Conclusion:**

L'analyse de profil clinique des traumatismes alvéolo-dentaires aux cliniques universitaires de Kinshasa est celui d'un adulte jeune de sexe masculin, présentant des luxations dentaires des maxillaires dont les causes sont les agressions/rixes.

## Introduction

Le traumatisme maxillo-facial peut être isolé ou associé à d'autres localisations [1], présentant des lésions tégumentaires, squelettiques ainsi que les traumatismes alvéolo-dentaires (TAD). Les traumatismes alvéolo-dentaires sont difficiles à appréhender, très souvent associés aux traumatismes maxillo-faciaux, mais peuvent aussi être isolés ce qui explique la difficulté des études épidémiologiques [1]. Certes il est évident qu'il existerait une sous-estimation importante de l'incidence et de la prévalence de ces traumatismes parce que, d'une part les patients viennent difficilement consulter, et d'autre part, par le fait que lors de la prise en charge d'un polytraumatisé, les lésions dentaires semblent ne pas être une priorité. Néanmoins ces affections restent aussi fréquentes. TAD représente 10% chez les adolescents selon Gineste [2], et 13,6% des enfants âgés de 6 à 15 ans selon Delattre [3]. Pour Gassner [4], la fréquence de ces traumatismes diminue avec l'âge car elle concerne 50% des enfants avant 10 ans contre 30% des 10-30 ans. Et Selon l'étude Adriano [5], la fréquence de TAD est de 10,8%. Le sexe masculin présente une prédisposition aux traumatismes dento-alvéolaires des dents permanentes que le sexe féminin avec le sexe ratio variant entre 1,3 à 2,3 [6]. Ces traumatismes touchent surtout l'homme jeune victime d´une agression/rixe, d'un accident de la voie publique ou d'une activité sportive [7-10]. Pour certains auteurs; en Chine [11], et au Canada [12], les accidents du trafic routier seraient la principale cause des traumatismes de la face y compris alvéolo-dentaire; par contre en Tanzanie [13], et en France [14], ils concluent que les agressions et les activités sportives étaient respectivement l'étiologie la plus répandue. En République Démocratique du Congo, en général et aux Cliniques universitaires de Kinshasa en particulier, l'épidémiologique des traumatismes alvéolo-dentaires reste encore méconnue. La présente étude rétrospective sur une période (7 ans), rapporte le profil épidémiologique des traumatismes alvéolo-dentaires afin de contribuer à une meilleure connaissance de ces lésions et à une prise en charge appropriée.

## Méthodes

Une étude transversale de 7ans a été réalisée dans le Département d'odontostomatologie, Service de stomatologie et de chirurgie maxillo-faciale, des Cliniques Universitaires de Kinshasa, en R.D.C allant de 2007 à 2014. La taille de l'échantillon a été de 287 dossiers de patients. Les critères d'exclusion étaient les dossiers de patients présentant les fractures de base osseuse maxillo- mandibulaire ou ayant des lésions des tissus mous seulement, les dossiers de patients qui n'ont pas fourni des informations complètes. Les dossiers de patients décédés avant l'évaluation initiale à l'Hôpital et ceux qui n'ont pas été rappelés selon le plan de traitement tel que détaillés dans notre première partie de cette étude ont été aussi exclus (Nyimi, Adelin et al 2017) [15]. Après exclusion de ces cas, 93 dossiers de patients ont participés à l'étude. Un formulaire de pré-enquête a été préparé à cet effet pour recueillir des données. Toutes les données démographiques telles que l'âge et le sexe du patient ont été recueillies. Les registres de consultation et les dossiers médicaux des patients ont été examinés afin d'extraire des informations relatives aux variables nécessaires telles que la cause du traumatisme, les dents traumatisées, le mode de blessure, les sites de fracture, et les modalités de traitement et le résultat. Le mode de traumatisme a été classé comme un accident de la circulation routière (ACR), agression/rixe, une chute de hauteur, une activité professionnelle, des sports, et la mastication. Les fractures alvéolo-dentaires ont été évaluées selon qu'il s'agit d'une contusion dentaire, fracture dentaire (coronaire, radiculaire ou corono-radiculaire), et subluxation dentaire, luxation dentaire partielle, luxation dentaire totale ou avulsion. Les fractures ont été prise en charge par plusieurs procédées thérapeutiques, notamment des extractions dentaires, Immobilisation avec Ligature en barres Arche d'Erich avec une fixation intermaxillaire par des fils et élastiques, soit une Immobilisation avec ligature des barres d'arc d'Erich sans fixation intermaxillaire et a de fois seule une observation. Les fractures alvéolo-dentaires ont été diagnostiquées par des radiographies retro-alvéolaires, profil défilé mandibulaire, face basse et panoramiques. Des statistiques descriptives avec moyenne et écart-type ont été utilisées. Le Test T-Student a été effectué pour comparer les variables entre les sexes. Le test du chi carré a été effectué pour évaluer l'association entre les variables qualitatives et quantitatives. Le niveau de signification a été fixé à 0,05, SPSS version 21 (SPSS Inc, Chicago, IL, USA) a été utilisé pour l'analyse statistique.

## Résultats

**Figure 1:**
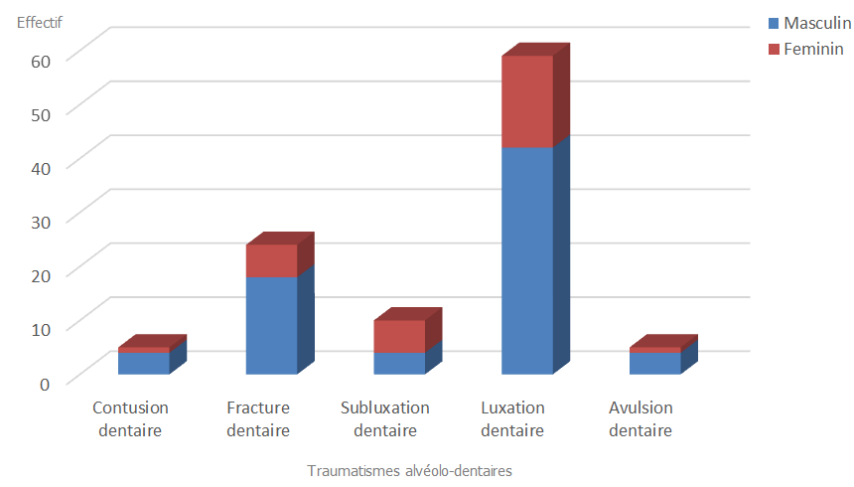
Répartitions des traumatismes alvéolo-dentaires en fonction du sexe

**Figure 2:**
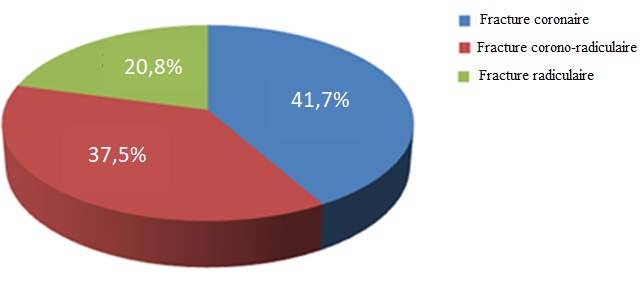
Répartitions de type de fracture dentaire

**Figure 3:**
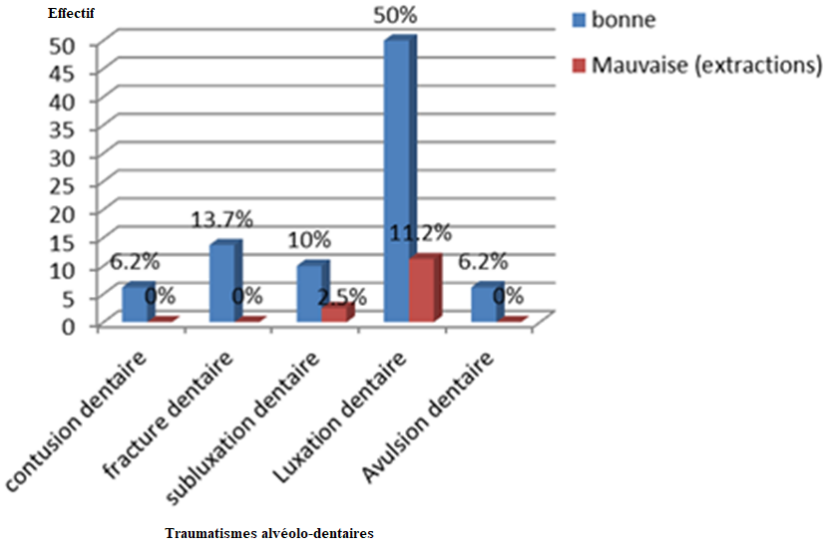
Traumatismes alvéolo-dentaires et évolution du traitement

Il ressort de nos résultats que les hommes sont le plus touchés par le traumatisme alvéolo-dentaire soit 66,7% de cas et la luxation dentaire a été prédominante soit 52,7% de cas (Figure 1). La fracture coronaire est le type de fracture dentaire la plus rencontrée (41,7%) suivis de la fracture corono-radiculaire (37,5%) (Figure 2). La tranche d'âge entre 20-29 ans était la plus touchée dans notre série soit 43% de cas (Tableau 1). Les agressions/rixes ont été les principales causes des traumatismes alvéolo-dentaires, soit 49/93 de cas (Tableau 2). L'incidence radiographique retro-alvéolaire a été la plus réalisée dans 75,7% de cas. Les incisives sont les dents les plus atteintes dans notre série et particulièrement les incisives supérieures, soit 55% (Tableau 3). Le blocage mono-maxillaire fut le traitement le plus réalisé avec 61,2% de cas (Tableau 4). La réussite du traitement a été observé dans 86,2% de cas (Figure 3).

**Tableau 1:** Distribution des traumatismes alvéolo-dentaires par tranche d’âge

Tranche d’âge (ans)	Traumatismes alveolo-dentaires					Total (%)
	contusion dentaire	fracture dentaire	subluxation dentaire	Luxation dentaire	Avulsion dentaire	
0-9	0(0)	3(3,2)	1(1,1)	2(2,1)	0(0)	6(6,4)
10-19	1(1,1)	5(5,4)	6(6,4)	8(8,6)	1(1,1)	21(22,6)
20-29	3(3,2)	8(8,6)	2(2,1)	25(26,9)	2(2,1)	40(43)
30-39	0(0)	4(4,3)	1(1,1)	7(7,5)	2(2,1)	14(15)
40-49	1(1,1)	2(2,1)	0(0)	2(2,1)	0(0)	5(5,4)
50-59	0(0)	1(1,1)	0(0)	3(3,2)	0(0)	4(4,3)
plus 60	0(0)	1(1,1)	0(0)	2(2,1)	0(0)	3(3,2)
**Total**	5(5,4)	24(25,8)	10(10,7)	49(52,7)	5(5,4)	93(100)

%: pourcentage

**Tableau 2:** Traumatismes alvéolo-dentaires et étiologies

Etiologies	Traumatismes alveolo-dentaires					Total (%)
	contusion dentaire	fracture dentaire	subluxation dentaire	Luxation dentaire	Avulsion dentaire	
Piéton	0(0)	0(0)	0(0)	0(0)	0(0)	0(0)
Véhicule	3(3,2)	5(5,4)	3(3,2)	10(10,7)	1(1,1)	22(23,6)
Moto	0(0)	2(2,1)	0(0)	3(3,2)	0(0)	5(5,4)
Aggression/rixes	2(2,1)	11(11,8)	3(3,2)	30(32,2)	3(3,2)	49(52,7)
Acc domestique	0(0)	3(3,2)	1(1,1)	1(1,1)	0(0)	5(5,4)
Acc de travail	0(0)	0(0)	0(0)	1(1,1)	0(0)	1(1,1)
Acc de sport	0(0)	0(0)	3(3,2)	3(3,2)	1(1,1)	7(7,5)
Mastication	0(0)	3(3,2)	0(0)	1(1,1)	0(0)	4(4,3)
**Total**	5(5,4)	24(25,8)	10(10,7)	49(52,7)	5(5,4)	93(100)

Acc: accident, %: pourcentage

**Tableau 3:** Distribution des traumatismes alvéolo-dentaires en fonction des dents traumatisées

Dents traumatisées	Traumatismes alveolo-dentaires					Total (%)
	contusion dentaire	fracture dentaire	subluxation dentaire	Luxation dentaire	Avulsion dentaire	
Incisive Sup	3(1,9)	20(12,5)	20(12,5)	41(25,6)	4(2,5)	88(55)
Canine Sup	1(0,6)	8(5)	3(1,9)	1(0,6)	0(0)	13(8,1)
Premolaire Sup	0(0)	1(0,6)	0(0)	1(0,6)	0(0)	2(1,2)
Molaire Sup	0(0)	1(0,6)	0(0)	1(0,6)	0(0)	2(1,2)
Incisive Inf	1(0,6)	10(6,2)	7(4,4)	20(12,5)	1(0,6)	39(24,4)
Premolaire Inf	0(0)	2(1,2)	0(0)	2(1,2)	0(0)	4(2,5)
Molaire Inf	0(0)	2(1,2)	0(0)	3(1,9)	0(0)	5(3,1)
Canine Inf	0(0)	5(3,1)	0(0)	2(1,2)	0(0)	7(4,4)
**Total**	5(3,1)	49(30,6)	30(18,7)	71(44,4)	5(3,1)	160(100)

Sup = supérieur, inf = inferieure; % = pourcentage

**Tableau 4:** Traumatismes alvéolo-dentaires et type de traitement réalisé

Traitements	Traumatismes alveolo-dentaires				Total (%)
	contusion dentaire	fracture dentaire	subluxation dentaire	Luxation dentaire	
Observation	5(5,7)	0(0)	0(0)	0(0)	5(5,7)
Extraction	0(0)	11(12,5)	1(1,1)	0(0)	12(13,6)
Transfert en D.O /protheses	0(0)	13(14,8)	0(0)	0(0)	13(14,8)
BIM	0(0)	0(0)	1(1,1)	4(4,5)	5(5,7)
BMM	0(0)	0(0)	8(9,1)	45(51,1)	53(60,2)
**Total**	5(5,7)	24(27,3)	10(11,4)	49(55,7)	88(100)

**Légende**: BIM = blocage intermaxillaire, BMM = blocage mono-maxillaire, D. O = dentisterie opératoire, % = pourcentage

Le blocage mono-maxillaire était le traitement le plus réalisé avec 60,2% de cas

## Discussion

Bien que les traumatismes alvéolo-dentaires (TAD) sont difficiles à appréhender, il ressort de notre étude que les hommes ont été plus touchés que les femmes soit (66,7%) de cas. Nos résultats sont similaires à ceux trouvés par d'autres auteurs [6-10]. Cette prédominance masculine trouverait probablement une explication par le caractère plus agressif et le goût du risque que possèdent les sujets de sexe masculin. Les hommes se coucheraient plus tard que les femmes et n'ont souvent pas peur de traverser des zones réputées dangereuses. La luxation dentaire était la forme de TAD la plus rencontrée dans la présente étude (52,7%); similaire aux résultats de l'étude d'Andrianony [5] et les fractures dentaires occupaient le second rang (25,8%). Parmi ces fractures dentaires, les fractures coronaires sans exposition de la chambre pulpaire étaient les plus rencontrées soit 41,7% de cas. Ces résultats concordent avec ceux trouvés par Herve Virginie [16] expliquant que les fractures coronaires sans exposition pulpaire sont les plus rencontrées sur les dents permanentes. La prédominance des luxations dentaires serait due au fait que lors des agressions/rixes il y a une fréquente concomitantes des lésions des tissus mous. Il est possible qu'un bouclier à lèvres “protégeant” ait pu absorber une grande partie de la force de l'impact du trauma et aurait pu répartir la force dans une zone dentaire plus grande. Par contre si un projectile est frappé directement sur une incisive supérieure on observera plus des lésions dentaires. Les TAD sont fréquent chez les sujets compris entre 10 ans et 29 ans avec un pic élevé dans la tranche d'âge entre 20-29 ans. Les présents résultats sont similaires à ceux de certains auteurs [4]. La grande activité physique et professionnelle à cette période de la vie, la recherche d'emplois et participation à des activités extérieures plus vulnérables par rapport aux autres groupes d'âge expliquerait ce pic élevé dans cette tranche âge. Par contre certains auteurs ont trouvés que les TAD toucheraient plus les enfants et les adolescents: Berkowitz et col [17] ont identifié trois périodes de la vie où l'on observe un taux élevé de TAD; une première période se situe entre l'âge de 1 et 3 ans, une deuxième entre l'âge de 7 et 10 ans et une troisième période se situe entre l'âge de 16 et 18 ans [17].

Pour Delattre [3], les TAD concerneraient les enfants de 6 à 15 ans et en fin pour Gassner [18] les TAD concerneraient 50% des enfants avant 10 ans contre 30% des 10-30 ans. L'explication qu'on peut fournir de ces observations des auteurs susmentionnés en termes de chaque groupe d'âge serait: 1) Jusqu'à 3 ans: est une période de la formation d'un os alvéolaire peu dense et peu minéralisé, donc malléable, favorisant les luxations des organes dentaires plutôt que les fractures; 2) De 3-6 ans: l'enfant acquiert la vitesse de déplacement, l'âge de la scolarité, l'os alvéolaire reste malléable, les attaches parodontales lâches et la racine est raccourcie par la rhizalyse physiologique; ce qui privilégient les luxations dentaires; 3) De 6 et 12 ans: l'édification radiculaire en cours confère quelques spécificités: malgré l'os alvéolaire devient compact et résistant aux déplacements latéraux et axiaux, la racine courte permet les luxations; 4) De 12 ans et plus: denture définitive édifiée et les conséquences d'un TDA sont plus lourdes. Tous les facteurs de risque liés à cet âge et ceux iatrogènes semblent générer le plus d'accidents. Les agressions/rixes ont été les principales causes des TAD dans notre étudie (52,7%) suivi des ATR (29%) principalement les véhicules (23,6%). Ce taux élevait d'agressions/rixes se justifierait par le fait qu'actuellement dans la ville province de Kinshasa, il ya l'insécurité orchestrée par le phénomène Kuluna et l'augmentation du taux de chômage, entrainerait des jeunes à se réfugier dans la prise des drogues et alcools, d'où l'augmentation du nombre d'agression. Nos résultats corroborent à ceux de Herve Virginie [16] selon laquelle Chez les jeunes adultes, les agressions/rixes restent l'étiologie principale. Bien que les ATR constituent 29%, soit la deuxième cause de la TAD, nous argumentons que ses résultats serait dû à l'imprudence des piétons et le fait que les conducteurs ont tendance à rouler très vite et ils ont du mal à freiner dès qu'un obstacle survient. En outre la défectuosité des freins des véhicules et le manque d'utilisation des ceintures de sécurité très souvent seraient les facteurs favorisants. Du fait que le maxillaire a une position plus antérieure que la mandibule, constitue un os le plus poreux et un pare-chocs naturel du visage, cela semble corollaire avec une atteinte beaucoup plus importante que celle de la mandibule (65,5% de TAD).

En raison de la documentation approfondie de ces fractures, comprenant à la fois l'information clinique, des radiographies et des photographies cliniques, il est commode d'établir leurs diagnostics avec précision. Aujourd'hui, avec des outils de diagnostic les plus avancés et disponibles, tels que CBCT, il est possible d'avoir une image précise de la fracture dans les trois dimensions et la précision du diagnostic est beaucoup plus élevée [19]. L'incidence radiographique retro-alvéolaire a été malheureusement la plus réalisée et la plus accessible à 76% chez les patients admis dans notre hôpital et cela au fait que la majorité des patients dans les pays en voie de développement en général et en RDC en particulier sont des patients à faible revenu. En dépit du fait que l'incidence Retro-alvéolaire ne pas à comparer avec le CBCT, ou soit appelé par certaines auteurs le cliché de débrouillardise; cette incidence donne aussi une bonne visualisation de l'image des parodontes, de la dent entière avec possibilité de diagnostiquer des fractures alvéolo-dentaires. Le blocage mono-maxillaire à IVY était le plus réalisé soit (66%). La pratique du BMM comme traitement de référence dans notre milieu, expliquerait non seulement que ce type de contention a l'avantage d'être rapidement réalisé, mais aussi et surtout qu'il nécessite que peu de matériel pour son utilisation. Actuellement, cette procédée est abandonné en raison de ses multiples inconvénients; notamment de desserrage spontané, des lésions iatrogènes de la gencive et tendance à l'égression des dents réimplantées. De nos jours le développement des techniques d'adhésion amélaire a permis de faire évoluer considérablement les techniques de contention. Blocage mono-maxillaire par arc de Duclos était réalisé dans 34% de cas, et a donné des bons résultats. L'observation comme attitude thérapeutique dans notre série a été faible soit 5,7%, comparée à celle trouvée dans les traumatismes maxillo-faciales (36,23%) par Nyimi et Col. [15]. Cette différence serait dû au fait que la prise en charge des fractures maxillo-faciales demanderait beaucoup d'exigence à savoir: un personnel qualifié et un plateau technique bien équipé. Par contre la prise en charge chirurgicale des TAD, semblerait être plus aisée et abordable par tous les praticiens, les matériels et matériaux chirurgicaux seraient facilement accessibles et un pronostic vital beaucoup plus favorable que ceux des traumatismes maxillo-faciaux.

## Conclusion

Le profil épidémio-clinique des traumatismes alvéolo-dentaires aux Cliniques Universitaires de Kinshasa est celui d'un adulte jeune (20-29 ans) de sexe masculin, présentant des luxations dentaires comme principale forme de traumatisme alvéolo-dentaire, causé par des agressions/rixes. Bien que l'incidence radiographie retro-alvéolaire aie était la plus réalisée et le blocage mono-maxillaire à ivy soit le traitement de choix, le CBCT et la technique d'adhésion amélaire demeurent indispensable dans le diagnostic et la prise en charge des traumatismes alvéolo-dentaires.

### Etat des connaissances actuelle sur le sujet

- La fréquence épidémiologique de ces traumatismes alvéolo-dentaire est étudiée dans plusieurs régions du monde;
- Les causes et les facteurs susceptibles influençant ces traumatismes;
- L'âge et le sexe de prédilection sont connus ailleurs.

### Contribution de notre étude à la connaissance

- Notion de la variabilité de la fréquence des TAD et de la cause selon le pays, la région et selon les coutumes (agression, 20-29 ans en RD. Congo). Ceci signifie que la méconnaissance de cette notion a peut-être fait passer inaperçu le diagnostic de TAD par les praticiens et en conséquence instauration de traitement inadéquat;
- Cette étude, constitue une référence pour toutes les études ultérieures de TAD en RD Congo.

## Conflits d’intérêts

Les auteurs ne déclarent aucun conflit d'intérêts.

## Remerciements

Nous remercions Monsieur Steve Sekele Masin pour sa contribution énorme dans ce travail avec les analyses des données statiques. Adelin Nzudjom Foche et Nyimi Bushabu Fidele ont travaillé équivalemment
